# Supplementary material for: A Comprehensive Assessment of Ultraviolet-Radiation-Induced Mutations in Flammulina filiformis Using Whole-Genome Resequencing
Source: J Fungi (Basel). 2024 Mar 20;10(3):228. doi: 10.3390/jof10030228 (PMC10971301; doi:10.3390/jof10030228)
Supplement: Supplementary file 1 [file jof-10-00228-s001.zip › Supplementary Material S8/KEGG annotation/out/64381550635650.os/KO/out_map/map04722.html]

KEGG PATHWAY: Neurotrophin signaling pathway - Reference pathway


|  |  |
| --- | --- |
| **Neurotrophin signaling pathway - Reference pathway** |  |

[
Pathway menu
| Organism menu
| Pathway entry
| Show description
| User data mapping
]

Neurotrophins are a family of trophic factors involved in differentiation and survival of neural cells. The neurotrophin family consists of nerve growth factor (NGF), brain derived neurotrophic factor (BDNF), neurotrophin 3 (NT-3), and neurotrophin 4 (NT-4). Neurotrophins exert their functions through engagement of Trk tyrosine kinase receptors or p75 neurotrophin receptor (p75NTR). Neurotrophin/Trk signaling is regulated by connecting a variety of intracellular signaling cascades, which include MAPK pathway, PI-3 kinase pathway, and PLC pathway, transmitting positive signals like enhanced survival and growth. On the other hand, p75NTR transmits both positive and nagative signals. These signals play an important role for neural development and additional higher-order activities such as learning and memory.

|  |  |
| --- | --- |
| Reference pathway | 100% |
